# Defining and measuring motor imagery in children: mini review

**DOI:** 10.3389/fpsyg.2023.1227215

**Published:** 2023-08-16

**Authors:** Ghazala T. Saleem

**Affiliations:** Department of Rehabilitation Sciences, School of Public Health and Health Professions, State University of New York at Buffalo, Buffalo, NY, United States

**Keywords:** motor rehabilitation, motor learning, motor imagery, children, measurements, mental practice

## Abstract

Motor imagery (MI) is the ability to engage in the mental representation of a task consciously or automatically without generating a voluntary movement. While the construct of MI and its various dimensions have been comprehensively studied in adults, research remains limited in children. Children as young as 5 years old can engage in MI, and this engagement is crucial to their motor development and skill acquisition. Further, the degree of skill achievement is directly linked to MI responsiveness. Clinicians and researchers often measure MI responsiveness in children to facilitate skill development and retention. However, few measures exist that can appropriately assess MI responsiveness in children. To date, a focused review examining the MI dimensions in children as well as comparing the characteristics of MI measures in children is lacking, and thus a research gap exists. This paper examines past and current research describing MI ability in children from the theoretical, developmental, and neurological lens and systematically analyzes the properties of three widely used operations – the movement imagery questionnaire in children (MIQ-C), the Florida praxis imaginary questionnaire (FPIQ-C), and the mental chronometry paradigm (MCP) – to measure MI and its dimensions in children.

## Introduction

Motor imagery (MI) is a highly investigated construct in adults both neurophysiologically and behaviorally (Collet et al., 2011), and many operations currently exist to assess this ability in the adult population; however, MI in children remains poorly understood with few measurements quantifying this ability in the pediatric population (Kwekkeboom et al., 2000). Recent controlled studies in children have emphasized that MI is not only imperative to performing everyday tasks but may also be used to enhance motor function (Behrendt et al., 2021). For example, a 2019 neuroimaging study in ten children with unilateral cerebral palsy (UCP) (aged 9–14 years) and ten age-matched typically-developing (TD) children investigating explicit MI (conscious imagination) ability for grasping actions using the mental chronometry paradigm (MCP) found that both children with UCP and TD children demonstrated temporal consistencies between executed and imagined movements (Errante et al., 2019). Further, higher scores on MCP were directly linked to higher activation in brain areas involved in real actions (e.g., intraparietal sulcus and dorsal premotor cortex) (Errante et al., 2019). Of note, previous studies have shown that implicit MI (unconscious imagination) as measured by the hand laterality paradigm is impaired in children with UCP. Similarly, a 2021 study in 138 young boys (mean age, 10.13 years, SD = 0.65 years) investigating the effects of MI dominance (visual versus kinesthetic dominance) and attentional focus [internal focus (focus on the arm); external focus (focus on the ball)] on a tossing motor task found that the extent to which attentional focus affected the tossing task depended on MI dominance, that is, an increased visual imagery dominance resulted in greater motor learning for children who engaged in external attentional focus, while higher levels of kinesthetic dominance resulted in decreased motor learning for children who embraced external focus (Bahmani et al., 2021). Both Errante et al. (2019) and Bahmani et al. (2021) studies provide two key insights regarding MI mechanism and evaluation in children: (a) MI dimensions must be understood and appropriately used while providing MI training to improve motor learning, and (b) MI measurements incorporating relevant MI dimensions must be considered when assessing MI in children.

While past and recent scientific reviews have discussed the importance of understanding MI development in children and described the effects of MI training in healthy and neurological pediatric populations, they lacked both a description of MI dimensions most useful to the rehabilitation of motor disorders and an analysis of MI measurements in children (Spruijt et al., 2015; Behrendt et al., 2021). Additionally, a theoretical explanation of the workings of MI in children has been overlooked. Therefore, to address these research gaps, this review examines the multidimensional MI construct in children theoretically and in light of current and past developmental and neurological research while comparing the characteristics of measures that are currently being used to evaluate MI in children.

MI involves using sensorimotor information to rehearse a task in the working memory without producing any voluntary movement (Guillot and Collet, 2010). Young children, five and above, have been shown to produce mental images by integrating sensorimotor data efficiently (Funk et al., 2005; Molina et al., 2008; Caeyenberghs et al., 2009a; Frick et al., 2009; Saleem and Gillen, 2019). Three theories−simulation theory, bio-informational theory, and symbolic learning theory elucidate the mechanism of MI in children (Mowrer, 1961; Hecker and Kaczor, 1988; Saleem and Gillen, 2019).

The simulation theory proposes a neurological and behavioral parallel between the mental states in which the action is simulated and executed (Jeannerod, 2001). Adult neuroimaging and physiological studies have provided evidentiary support for this functional equivalence by using fMRI and autonomic feedback approaches (Decety, 1996; Guillot et al., 2010). Evidence of neurological similarities between the actual and virtual states of action have also been found in children using the mental chronometry paradigm (MCP) (Molina et al., 2008). In a classical study of 5–7 years old children (*n* = 80), similar movement time was noted across virtual and real conditions where children either imagined carrying a puppet to a targeted distance or actually carried the puppet to the targeted distance (Molina et al., 2008). As noted above, in a recent study by Errante et al. (2019), these findings were duplicated in twenty children (10 children with unilateral cerebral palsy (UCP) and 10 typically-developing children) where all children showed temporal congruities between imagined and real movement as demonstrated by MCP (Errante et al., 2019).

The bio-informational theory proposes a logical link between an abstract image and real world output by proposing a corresponding muscle activity with motor image generation (Hecker and Kaczor, 1988). Jean Piaget, the famous child psychologist, describes that around 2–7 years of age, children start engaging in imitation, imagery, and pretend play, and around 11 years, children start connecting the abstract representations with real-world outputs (Barrouillet, 2015). A study assessing movement representation in a sample of 58, 7–12 years old children using the radial pointing task (participants were instructed to move a pen to a circle drawn in the center of a piece of paper and then imagine moving the pen to the circle on the paper) and the mental rotation paradigm (a single stimulus of hand was presented on the screen, and the participants were asked to imagine rotating their hand until it looks the same as the stimulus presented on the screen) found that while children of all ages could engage in MI, the ability to connect the image with the motor output improved around 7–8 years of age (Caeyenberghs et al., 2009a). A recent 2020 study following the similar methodology of evoking images using the mental rotation paradigm in a sample of 164 children (aged 6–13 years) and 30 adults found similar outcomes as the Caeyenberghs et al. (2009a) study in that while six-year old children were capable of generating images, this ability improved with age, and children ten-year old and above demonstrated the same MI ability as adults (Souto et al., 2020).

The symbolic learning theory implies that symbol system assists with both image formation and task execution (Mowrer, 1961). Coded actions in the brain generate a blueprint to make the movement pattern familiar (Mowrer, 1961). A recent study by Saleem and Gillen (2019) in children aged 6–8 years old (*n* = 20) with handwriting dysfunction found that dysgraphia in children improved (as measured by the Minnesota Handwriting Assessment) over a period of 4 weeks (eight MI sessions total) when children systematically imagined alphabets alongside engaging in handwriting practice (Saleem and Gillen, 2019).

Notably, MI construct is multifaceted having both explicit and implicit dimensions (Nilsen et al., 2012; Suica et al., 2018). Explicit MI involves an active representation of a movement with conscious mental effort while implicit MI involves the unconscious or intrinsic imagination of a movement (Suica et al., 2018). Children 10 years and younger may benefit from implicit MI training; whereas, explicit MI training is more beneficial for children who are older than 10 years (Spruijt et al., 2015). Further, recent research has shown that children with UCP may also benefit from explicit MI training as opposed to implicit MI training (Errante et al., 2019).

**FIGURE 1:**
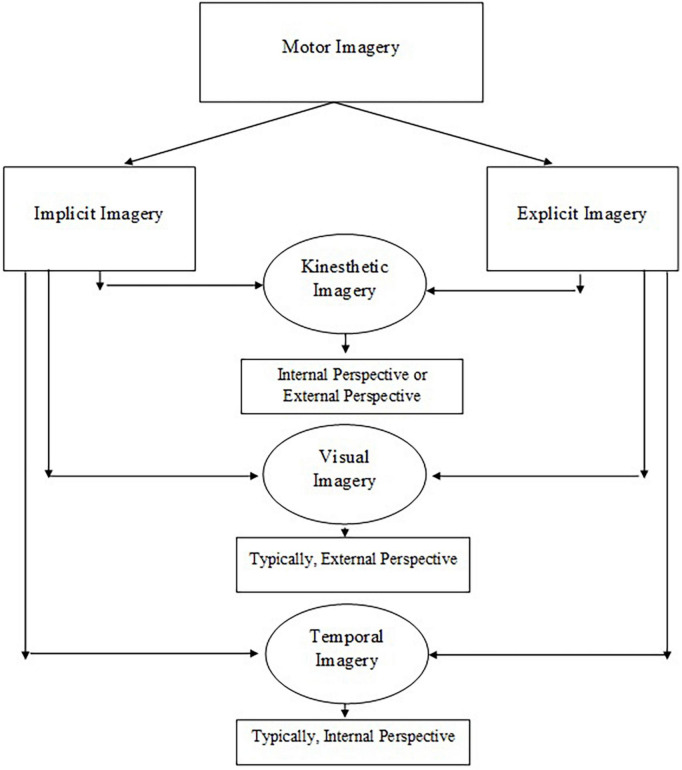
The construct of motor imagery is multidimensional including kinesthetic, visual, and temporal imagery.

Nested within the explicit and implicit dimensions are the kinesthetic, visual, and temporal dimensions of MI (Suica et al., 2018). The kinesthetic dimension requires children to feel the movement while having an awareness of body parts, position, and effort required to perform the movement (Martini et al., 2016). The visual dimension refers to how a child sees the movement while having an awareness of the space and size of the movement (Martini et al., 2016). The temporal dimension specifies the timing of the movement (Spruijt et al., 2015). Inherited within these three dimensions are two subdimensions of internal and external perspective (Martini et al., 2016). Internal (i.e., first-person) perspective denotes to performing the movement oneself and can represent kinesthetic, visual, or temporal imagery (Martini et al., 2016). Whereas, the external (i.e., third-person) perspective refers to watching the movement and usually involves the visual imagery dimension (Martini et al., 2016; Figure 1).

Motor imagery (MI) dimensions become essential when MI training is used to acquire or relearn motor skills (including reaching skills) in children (Gabbard et al., 2009; Saleem and Gillen, 2019). MI is an active component of motor planning (Bhoyroo et al., 2019), which is the ability to select a movement plan from an unlimited number of goal-oriented solutions (Bhoyroo et al., 2019). An appropriate movement plan is also crucial to estimating distance of an object from a specific body position (seated versus standing), and MI plays an integral role in judging whether an object is within or out of reach (Gabbard et al., 2009). Previously, several studies have used MI successfully to improve motor skills in children with movement dysfunction (Wilson et al., 2002; Rehbein and Doussoulin, 2011; Saleem and Gillen, 2019).

In a study of 7–12 years old children (*n* = 54) with developmental coordination disorder (a neurodevelopmental disorder defined by the difficulty in acquiring and performing age-appropriate coordinated skills, such as tying shoes and climbing stairs) both internal and external perspectives of MI were used to enhance motor coordination skills (Wilson et al., 2002). Children were divided into MI group, motor perceptual group, and a control group. Both children in MI group and motor perceptual group improved on motor skills tasks as measured by the standardized movement assessment battery questionnaire (MABQ), whereas the control group did not show any improvements (Wilson et al., 2002). This study was replicated by Wilson et al. (2016) in 30 children with DCD, and the results showed consistency with their earlier study in that both MI cohort and motor-perceptual group significantly improved on motor skills (as demonstrated by MABQ), whereas the results were insignificant for the control group. Similarly, another study used both internal and external MI dimensions to train sixty-four 9–10 years old children on throwing a ball to a distant target task (Rehbein and Doussoulin, 2011). Children received either MI training, modeling (recorded videos) training, or physical practice. The intervention efficacy was measured using the standardized basic and combined movement scale and the distance reached on each ball throw. Both children in MI and modeling groups improved on the throwing task. However, children in the physical practice group showed no improvement (Rehbein and Doussoulin, 2011). Another study conducted in 2014 studied the effects of physical practice and kinesthetic MI on the short- and long-term learning of the thumb opposition task in thirty-six 9–10 years old children. The children were divided into three groups, the physical practice group, the kinesthetic MI group, and the no-practice group. The outcome variable was the number of correct sequences per minute of the finger opposition task, and the physical practice group and the MI group were trained on the sequence of the task. All children were evaluated after 5 min, 4 days, 7 days, and 28 days. The results showed that while both the physical practice and MI group significantly improved on the trained sequence of the finger opposition task, only the MI group transferred this learning to a novel, untrained reverse sequence of the finger opposition task (Asa et al., 2014). A 2019 study used the internal or first-perspective MI in conjunction with physical practice in 6–8 years old children (*n* = 20) with handwriting dysfunction and found that children not only showed improvement in handwriting skills as measured by the Minnesota Handwriting Assessment when MI was added to the physical practice of handwriting but these improvements were also retained at the 1-week follow up testing (Saleem and Gillen, 2019).

Enhancing reaching skills in children is an essential area of motor rehabilitation. The kinesthetic dimension of MI, a fundamental aspect of motor planning, has provided an understanding regarding age-dependent effects on reaching estimates in children. A study investigating reaching estimates in 7-, 9-, and 11-years old children (*n* = 43) from an upright seated position and a more challenging standing on a single leg while leaning forward position showed that while children of all age groups slightly overestimated reach in both postural conditions, there were no significant effects of age or reaching condition on reaching estimates. However, when author compared the study results to a similar adult report, they found that the children’s overestimation values were greater than the adult overestimation values, indicating that reaching estimates may continue to refine with advancing age and that young children tend to overestimate their physical abilities (Gabbard et al., 2009; Cordova and Gabbard, 2014).

Appropriate assessment of imagery responsiveness is crucial to guide and optimize MI intervention in children. A study in twenty, 6–8 years old children showed that those children who were moderate to high imagers (as demonstrated by the kids imaging ability questionnaire) significantly improved on handwriting scores (as measured by the Minnesota Handwriting Assessment) compared to children who were low imagers (Saleem and Gillen, 2018).

To obtain information on MI measures that are currently being used in children, a systematic search of literature was conducted on “PubMed” and “Web of Science” using the terms “motor imagery” and “mental practice” and “children”; this search yielded 19 and 10 results, respectively. The abstracts of all the articles were reviewed. Three operations, namely the movement imagery ability questionnaire in children (MIQ-C), the Florida praxis questionnaire in children (FPQC), and the mental chronometry paradigm (MCP) were selected based on the literary evidence for these measures. After the selection of the instrument, three additional searches were conducted in PubMed, each with the name of the instrument and search terms including both “test reliability” and “test validity.” Most studies fitting the selection criteria were found on the FPIQ-C, followed by the MIQ-C, and the least number of articles were found on the MCP.

## Three operations measuring motor imagery in children

### The movement imagery questionnaire for children (MIQ-C)

The MIQ-C was adapted from the adult MIQ and assesses both the kinesthetic and visual dimensions of MI using internal and external perspectives (Martini et al., 2016). The MIQ-C has been used in research, clinical practice, and theory development. A study conducted in 93 children with or without developmental coordination disorder (DCD), aged 7–12 years old, showed that MIQ-C discriminated two groups on the kinesthetic subscale (Fuchs and Caçola, 2018). However, the overall differences in MI ability were not significant (Fuchs and Caçola, 2018). Another study testing an intervention (PETTLEP) to enhance the effects of imagery in 36 young athletes (mean age = 9.72, SD = 2.05) showed that children demonstrated no differences in the MI ability after the PETTLEP intervention on the MIQ-C (Quinton et al., 2014). One study assessing the effects of MI using the MIQ-C in 18 healthy children aged 9–10 years showed that MI combined with physical practice was more effective in improving mental task representation in children (Takazono et al., 2018).

The MIQ-C contains 12 items that use simple language and are suitable for children 7 years and older. Each item is noted on a 7-point Likert scale that anchors responses in a ranking order of difficulty: very hard/hard/kind of hard/not easy nor hard/kind of easy/easy/very easy. The MIQ-C can be completed and scored within 15–20 min. The test is scored by aggregating the responses on all 12 items, and higher scores denote to better performance.

The MIQ-C was validated in a sample of 206 children aged 7–12 years by using the confirmatory factor analysis approach. The factor loading correlations ranged from 0.51 to 0.69, the comparative fit index (CFI) was 0.93, and the root mean square error of approximation (RMSEA) was 07. The test-retest reliability, intraclass correlation coefficient (ICC) classifications, assessed in a sample of 23 children aged 7–12 years ranged from good for kinesthetic imagery (0.82), moderate for internal visual imagery (0.72), and acceptable for external visual imagery (0.43). Yavari Kateb et al. (2019) analyzed the construct validity and reliability of the Persian version of the MIQ-C in 135 children (7–12 years old) using factor analysis statistics and found the test to contain a 3-model solution comprising 12 items. The authors reported the CFI to be 0.98 and the RMSEA to be 0.057, indicating a good fit. The reliability of the found factors was reported to be good (Cronbach alpha = 0.85) (see Table 1).

**TABLE 1:** Reliability and validity information for the MIQ-C and the FPIQ-C.

References	Scale	Reliability estimates	Reliability estimate type	Validity estimates	Validity estimate type	Number of items	Sample	*N*
Martini et al., 2016	MIQ-C	0.82 (kinesthetic imagery); 0.72 (visual imagery); 0.43 external visual imagery	*ICC	0.93;0.07	**CFI; ***RMSEA	12	7–12 years old children	23
Yavari Kateb et al., 2019	MIQ-C (Persian version)	0.85	Cronbach’s alpha	0.98; 0.057	CFI; RMSEA	12	7–12 years old children	135
Chang and Yu, 2016	FPIQ-C	0.998 (praxis imagery); 0.981 (verbal gesture production); 0.991 (imitative gesture production); 0.995 (knowledge of object use)	ICC	0.965; 0.039	CFI; RMSEA	32 items	6–8 years old children	239

*Intraclass correlation coefficient.

**comparative fit index.

***root mean square error of approximation.

### The Florida praxis imagery questionnaire (FPIQ-C)

The Florida praxis imaginary test (FPIQ) for children (FPIQ-C) was adapted from the adult version of the FPIQ (Ochipa et al., 1997; Wilson et al., 2001) and assesses the ability to mentally picture complex movement tasks in children 6 years or older (Wilson et al., 2001). Specifically, the FPIQ-C assesses the ability to imagine the position of joint, the position of the body, and the spatial position of the object while performing skilled motor tasks (Wilson et al., 2001) and is used both in research and clinical practice. The FPIQ-C measure two direct dimensions of MI, namely kinesthetic and visual imagery and two linked dimensions of MI including verbal gesture production and imitative gesture production (Chang and Yu, 2018).

Several studies have shown that the FPIQ-C discriminates mental representation of learned motor tasks between children with DCD and healthy children (Wilson et al., 2001; Fuchs and Caçola, 2018). A study assessing the ability to mentally represent coordinated movements in 17 children aged 6–8 years old with DCD showed that children with DCD performed significantly lower (*p* = 0.003) on the FPIQ-C compared to children at risk with DCD and typically-developing controls (Chang and Yu, 2016). Another study evaluating the mental representation of skilled movements in 93 children with DCD (aged 7–12 years old) showed that children with DCD performed significantly lower on the FPIQ-C compared to typically-developing controls (Fuchs and Caçola, 2018). A study identifying representation of praxis in 20 children with DCD, aged 8–12 years old, showed that children with DCD performed significantly worse (*p* = 0.024) than healthy controls (Wilson et al., 2001). However, a study conducted in 22 children with cerebral palsy (CP), aged 5–9 years old, showed no significant differences in the ability of MI as measured by the FPIQ-C (Lust et al., 2016). This study concluded that children with CP may have implicit (automatic internal representation of motor tasks) difficulties versus explicit (instructed internal representation of motor tasks) difficulties on praxis tasks (Lust et al., 2016).

The FPIQ-C is an interview- and instruction-based scale and comprises 8-items in four subscales (kinesthetic, body position, action, and object) (Chang and Yu, 2016). The FPIQ-C is scored based on the correct answers (a correct answer is awarded 1 point) (Chang and Yu, 2016). A total of 32 correct answers are possible, and higher scores signify better performance on the FPIQ-C (Chang and Yu, 2016).

The reliability and construct validity of the FPIQ-C have been investigated in 239 Taiwanese school-age children (6–8 years old) with coordination or praxis disorders (Chang and Yu, 2018). Four models were evaluated, and the adjusted goodness of fit index (AGFI) was calculated. Model three displayed four dimensions, including kinesthetic position, verbal gesture, imitative gesture, and the knowledge of object use. All dimensions showed an excellent model fit with the lowest RMSEA (0.039) and highest CFI (0.965) (Chang and Yu, 2018). The concurrent validity analysis revealed a strong correlation (*r* = 0.836, *p* < 0.001) between the gesture subtests of the FPIQ-C and the gesture subtests of the sensory praxis integration test (SPIT) (Chang and Yu, 2018). The inter-rater reliability was also high for all subtests. Strong positive ICC correlations were observed (praxis imagery = 0.998, verbal gestural production = 0.981, imitative gestural production = 0.991, and knowledge of object-use = 0.995; all *p*-values < 0.001). The Cronbach alpha (0.836) demonstrated high internal consistency for the full FPIQ-C scale (Chang and Yu, 2018; Table 1).

### The mental chronometry paradigm

One of the most frequently used paradigms to study MI in children is the mental chronometry paradigm (MCP) (Spruijt et al., 2015). The MCP assesses if temporal congruence exists between the performance of an actual task and an imagined task (Spruijt et al., 2015). If the duration of the performance of a real and imagined task aligns, a high ability to participate in MI is denoted (Spruijt et al., 2015). In the MCP, the imagined movement is subjected to the same parameters or constraints as the real movement to ensure optimal participation in MI (Spruijt et al., 2015). The MCP is an instruction-based paradigm. Typically, a paper and pencil to record the time and a stopwatch are required.

Children age 5 and up can engage in the MCP (Spruijt et al., 2015). However, the ability to achieve temporal congruence between an actual and imagined task increases with age (Caeyenberghs et al., 2009b). Though most studies of mental chronometry have been undertaken in healthy children, it has also been shown that children with mild to moderate motor disorders and visual impairments can also participate in the MCP (Spruijt et al., 2015). However, considering the brain areas involved in MI, it can be safely assumed that children with posterior parietal cortex lesions may not partake in the MCP (Aflalo et al., 2015).

A mental chronometry time study conducted in three groups [healthy children, children with cerebral palsy (CP), and healthy adults] comprising 28 participants showed that while the duration of the actual and simulated time for healthy adults and children with CP did not correspond, healthy children aged 4–14 years old were able to significantly match their actual movement time with the simulated movement time (Iosa et al., 2014). The authors concluded that the differential walking pattern of children with CP might have contributed to insignificant results in these children (Iosa et al., 2014). Another study in 80 healthy children aged 5–7 years old demonstrated significantly higher congruence between an actual and virtual walking task that involved carrying and imagining carrying a heavy puppet to a targeted distance (Molina et al., 2008). In another study, three groups of children (6, 8, and 10 years old) and 22-year old adults virtually and actually performed a drawing task. Both children and adults robustly matched the duration of the virtual and actual drawing task (Skoura et al., 2009).

A study conducted in 30 elementary school children aged 7–8 years old and 60 middle school children aged 11–12 years old showed that the middle school children significantly corresponded on the duration of the performance of an actual obstacle course and an imagined obstacle course (Hoyek et al., 2009). Interestingly, boys showed a better temporal congruence compared to girls (Hoyek et al., 2009).

The MCP is being used in both clinical practice and research and assesses the temporal dimension of the MI construct. Visual and kinesthetic dimensions are typically not covered by the MCP. Despite the MCP’s widespread use to assess MI in children, the reliability and validity evidence for the MCP in children is lacking.

## Discussion

Though a highly research construct in adults, MI along with its various dimensions remains an understudied subject in children. Despite several benefits of MI training in children regarding skill development and acquisition, we only found a few recent studies examining, measuring, and using MI in children. Notably, this is the first review that has highlighted the importance of evaluating and applying the MI construct in children while characterizing and comparing the frequently used operations to measure MI in children.

As our review has shown, developmentally and neurologically children can engage in MI training at an early age. And thus, motor skill acquisition and rehabilitation could be facilitated using MI training in children. However, appropriate dimensions of MI must be used as interventions to enhance the therapeutic efficacy of the MI training. Additionally, relevant measures with good psychometric properties are required to evaluate MI in children so that individualistic care could be provided. Clinicians may provide imagery training to those who score high on imagery ability measures and may choose other appropriate interventions for children who score low on these measures.

A multimodal battery of MI must be used in children to comprehensively evaluate the MI ability as none of the widely used operations in children, which we studied, covered the multidimensional construct of imagery in its entirety. The MIQ-C and the FPIQ-C represented the kinesthetic and visual aspects, while the MCP embodied the temporal aspect. Though the MIQ-C has shown that it can reliably assess MI vividness in children, it can be further improved by adding content related to MI accuracy (spatial manipulation and orientation of objects) (Fuchs and Caçola, 2018). The MIQ-C could be an appropriate measure to assess MI ability in children who are within the age ranges of 7–12 years old based on its validity and reliability estimates; however, since these estimates were assessed in a small sample of children, future research is needed to verify psychometrics in a larger sample.

The FPIQ-C appears to be a valid and reliable test to assess the ability of MI in children aged 6–8 years old. However, more studies need to be conducted to replicate these findings in various age- and ethnicity/race-based samples of children for generalizability. Additionally, though the FPIQ-C has been shown to be responsive to MI accuracy in children with DCD, this scale may not successfully measure this ability in children with other motor disorders. Thus, the FPIQ-C needs to be used in other populations of children with motor disorders such as CP and/or traumatic brain injury (TBI) to validate the initial findings. Moreover, this scale may not be able to assess praxis imagery in children who have difficulty producing representations on command, which is an apparent weakness of this measure.

Though MCP appears to be more objective than some of the other MI measures discussed in this review, as children are blinded to the actual and virtual movement time, its psychometric properties need to be evaluated thoroughly. Further, this paradigm may be more suitable for older children than younger children as the ability to engage in the MCP improves with age. Boys may be more apt than girls to engage in the MCP (Dhouibi et al., 2021). Nonetheless, the ease of measurement and consistent results in children continue to allow researchers to use this paradigm to assess MI ability in children.

Lastly, as MI processes are covert, future neuroimaging studies must be conducted in children so that the correlations between the measures discussed in this paper and brain data can be computed. These correlations may provide a better understanding about the utility of the MI measures and could be helpful in identifying children that respond better to MI training.

## Author contributions

GS conceptualized, wrote, edited the manuscript, and approved the submitted version.
